# Supplementary material for: Control of non-homeostatic feeding in sated mice using associative learning of contextual food cues
Source: Mol Psychiatry. 2018 Jun 6;25(3):666–79. doi: 10.1038/s41380-018-0072-y (PMC6281813; doi:10.1038/s41380-018-0072-y)
Supplement: Supplementary file 1 — Supplementary Figure 1 [file 41380_2018_72_MOESM1_ESM.pdf]

a

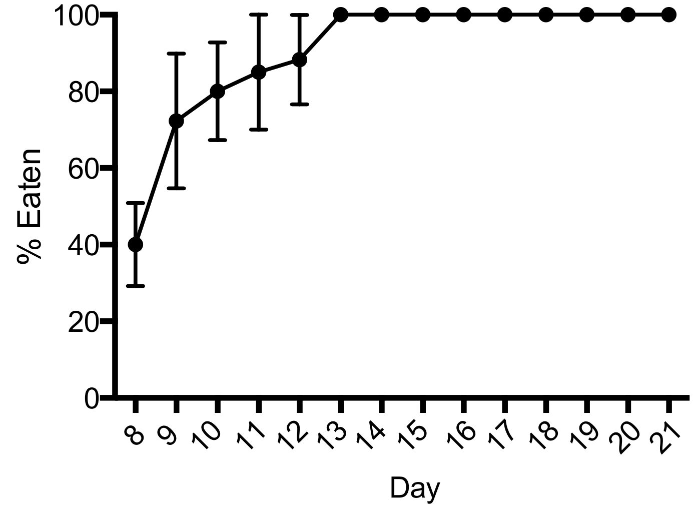

b

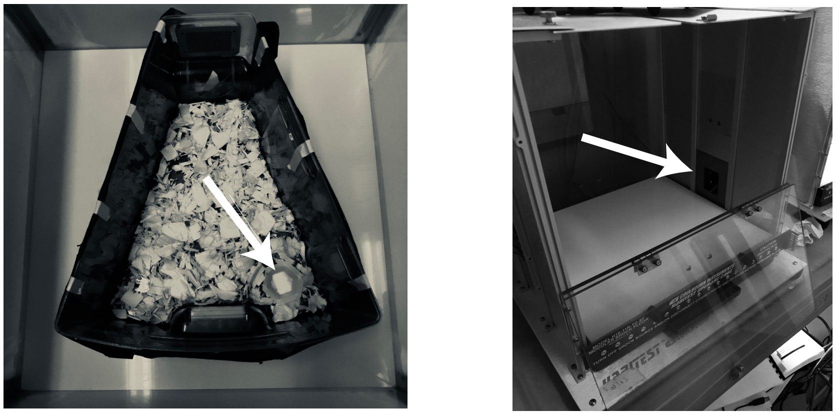

Supplementary Figure 1: Pellets eaten during Cued-IF and photos of Ctx-IF contexts.
a. Graph depicts the percentage of the total pellets distributed during training sessions that were retrieved and eaten. After the fifth session, mice eat 100% of the pellets given during the session.
b. Photos of the two contexts used to establish context-induced feeding. Contexts clearly differed in shape, floor texture, and position/type of food well. Contexts were also placed in different rooms. Arrows indicate the position of the food well in each context.

Stern Supplementary Fig 1
