# Supplementary material for: Control of non-homeostatic feeding in sated mice using associative learning of contextual food cues
Source: Mol Psychiatry. 2018 Jun 6;25(3):666–79. doi: 10.1038/s41380-018-0072-y (PMC6281813; doi:10.1038/s41380-018-0072-y)
Supplement: Supplementary file 2 — Supplementary Figure 2 [file 41380_2018_72_MOESM2_ESM.pdf]

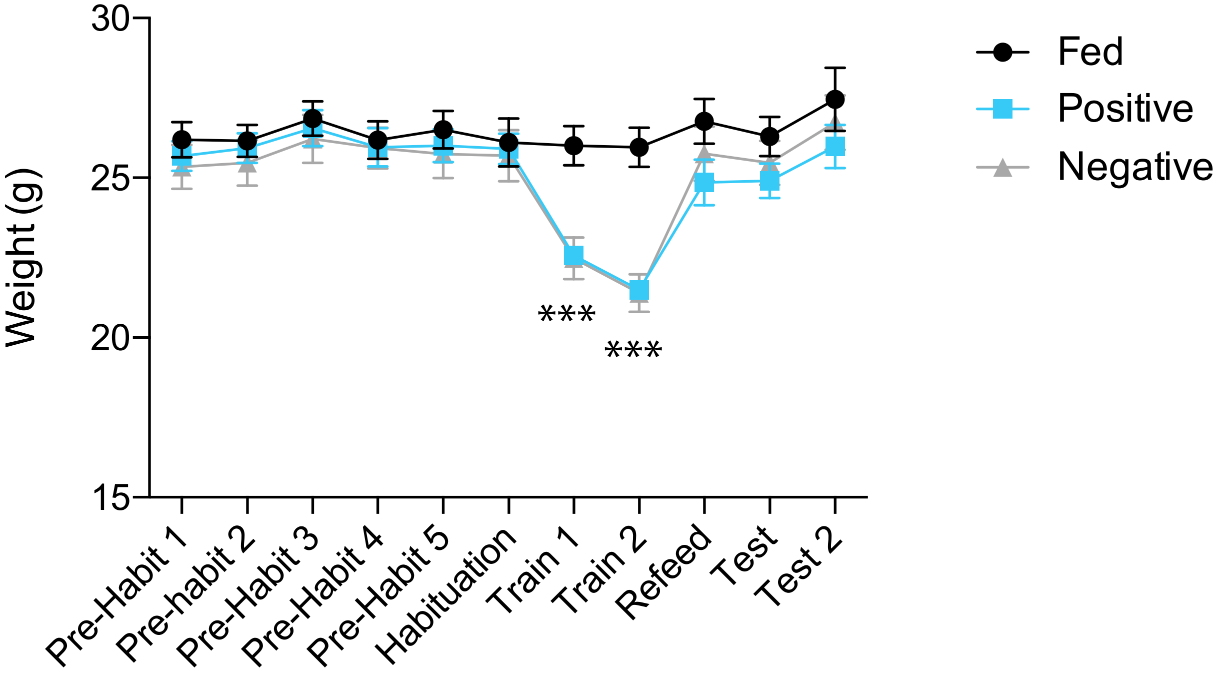

Supplementary Figure 2: **Comparable weights during Ctx-IF testing.**
Both the positive and negative valence groups show decreased weight, compared to fed controls, during training sessions when they have been fasted beforehand. Weights of all groups are comparable during Test 1 (48h after the last training session) and test 2 (1 week after Test 1).

Stern Supplementary Fig 2
