# Supplementary material for: Control of non-homeostatic feeding in sated mice using associative learning of contextual food cues
Source: Mol Psychiatry. 2018 Jun 6;25(3):666–79. doi: 10.1038/s41380-018-0072-y (PMC6281813; doi:10.1038/s41380-018-0072-y)
Supplement: Supplementary file 3 — Supplementary Figure 3 [file 41380_2018_72_MOESM3_ESM.pdf]

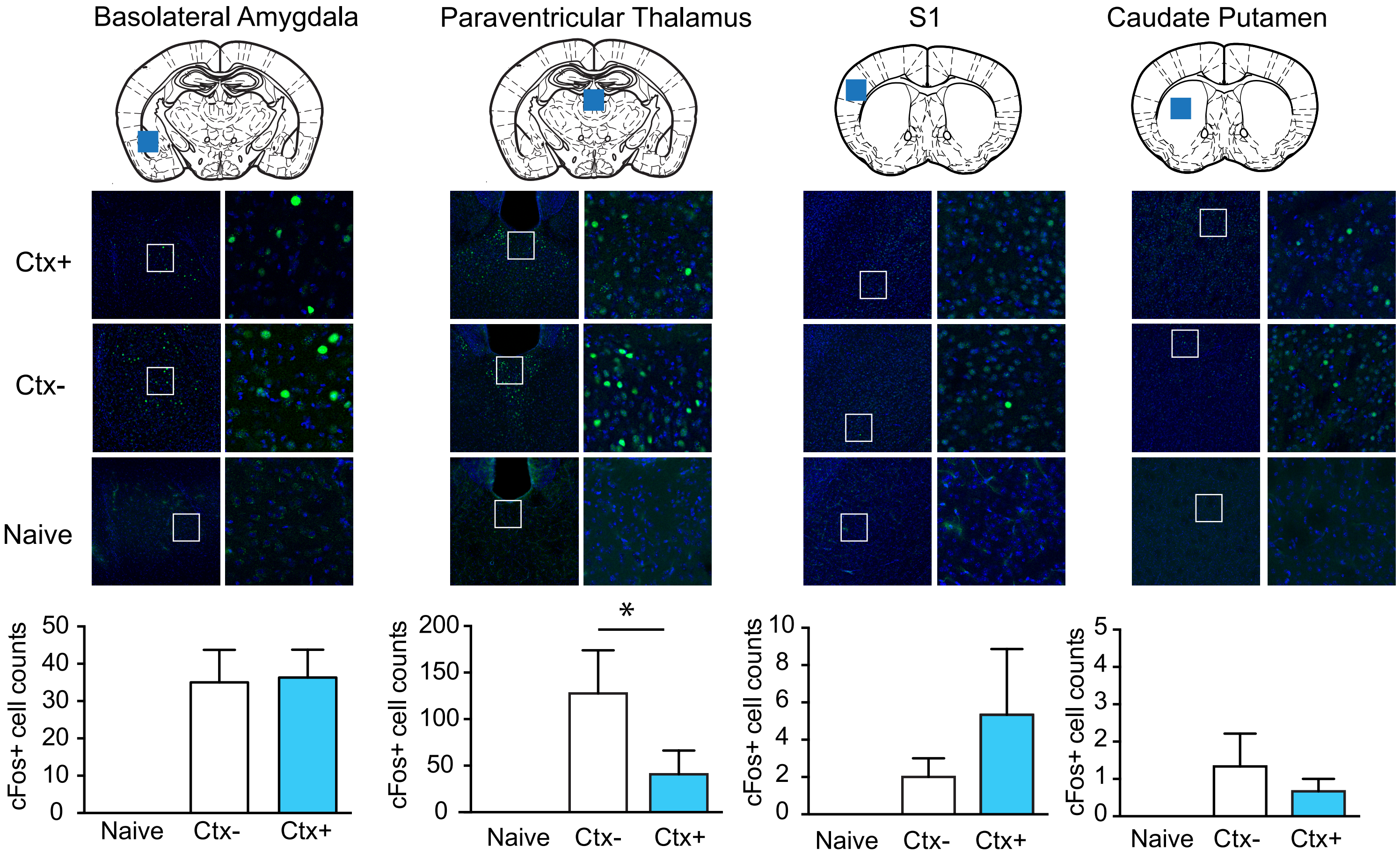

Supplementary Figure 3: Cfos staining in various brain regions.
cFos+ cells were counted in both Ctx+ and Ctx- animals in various brain regions. The basolateral amygdala showed equivalent numbers of cFos+ cells in both conditions, whereas the Ctx- contained more cFos+ cells than the Ctx+ group in the paraventricular thalamus. Both S1 and the caudate putamen showed very few cFos+ cells in both conditions.
