# Supplementary material for: Control of non-homeostatic feeding in sated mice using associative learning of contextual food cues
Source: Mol Psychiatry. 2018 Jun 6;25(3):666–79. doi: 10.1038/s41380-018-0072-y (PMC6281813; doi:10.1038/s41380-018-0072-y)
Supplement: Supplementary file 4 — Supplementary Figure 4 [file 41380_2018_72_MOESM4_ESM.pdf]

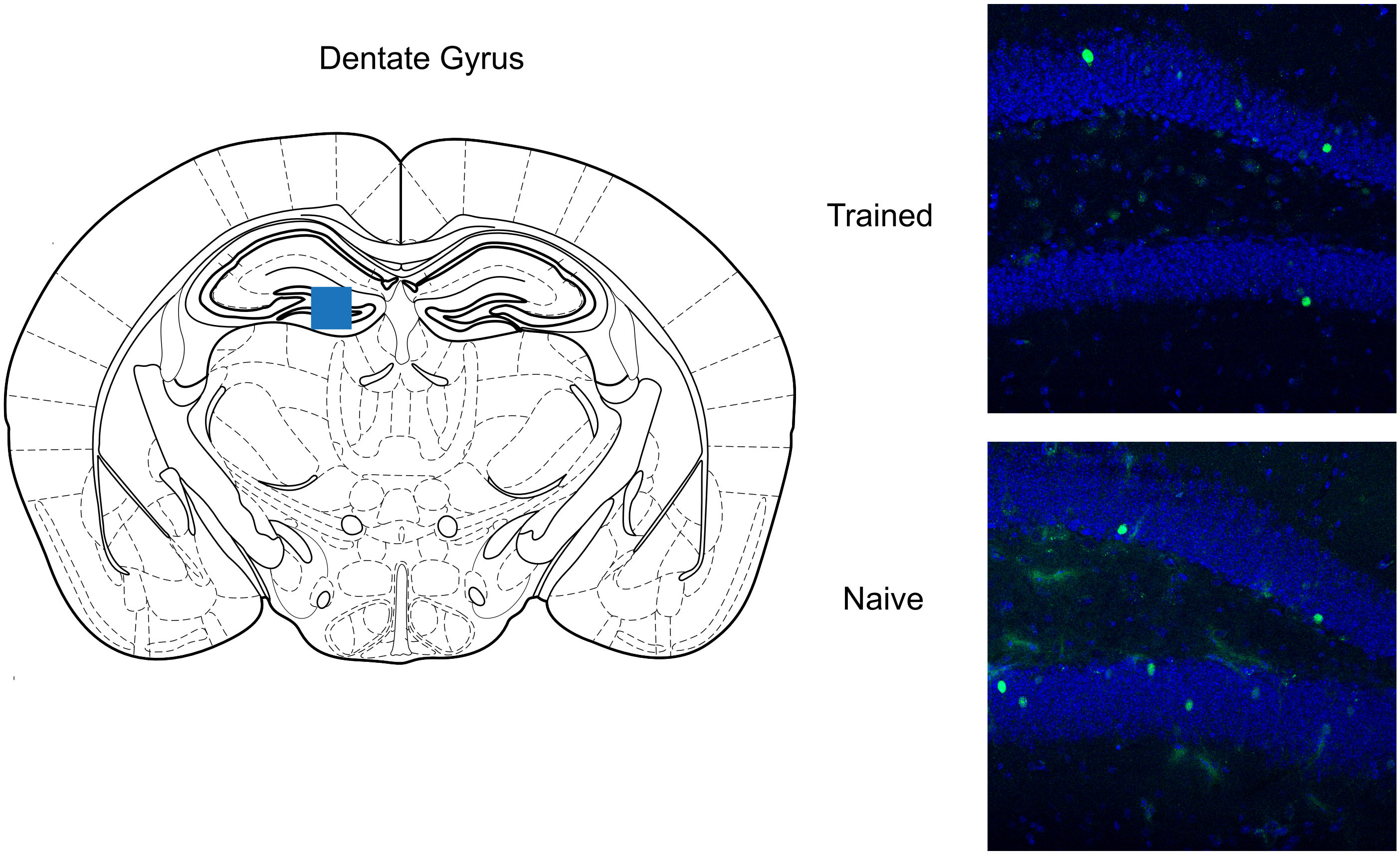

Supplementary Figure 4: Cfos staining in the dorsal hippocampus.
Both Naive and Trained mice display activated cells, as measured by Cfos (green), in the hippocampus following ctx-IF testing (top) compared to Naïve mice (bottom). DAPI is stained in blue.

Stern Supplementary Fig 4
