# Supplementary material for: Control of non-homeostatic feeding in sated mice using associative learning of contextual food cues
Source: Mol Psychiatry. 2018 Jun 6;25(3):666–79. doi: 10.1038/s41380-018-0072-y (PMC6281813; doi:10.1038/s41380-018-0072-y)
Supplement: Supplementary file 5 — Supplementary Figure 5 [file 41380_2018_72_MOESM5_ESM.pdf]

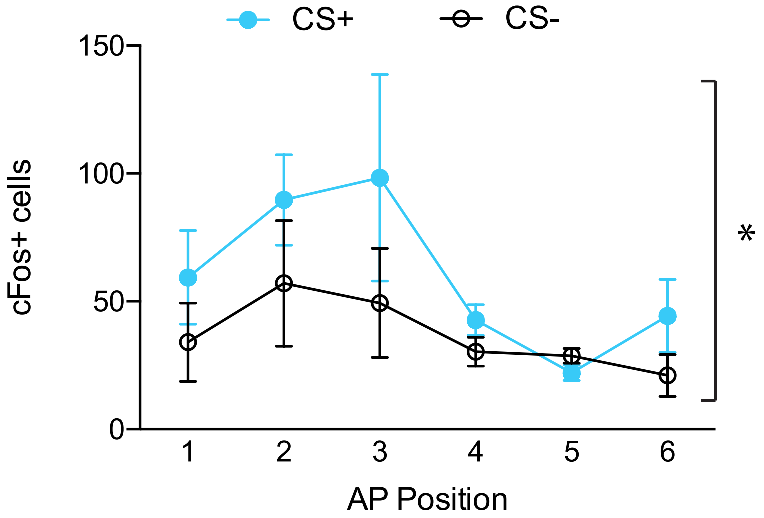

Supplementary Figure 5: Detailed analysis of cFos within the insular cortex
cFos+ cells were counted in both Ctx+ and Ctx- animals along 6 A/P axis positions ranging from +2.1mm to -1.06mm relative to bregma. This analysis revealed that although cFos+ counts were significantly increased overall in the Ctx+ group compared to the Ctx- groups, there was no specific subregion along the A/P axis that revealed a significant difference.

Stern Supplementary Fig 5
